# Surface Modification of Silk Fabric by Polysaccharide Derivatives towards High-Quality Printing Performance Using Bio-Based Gardenia Blue Ink

**DOI:** 10.3390/ma17143611

**Published:** 2024-07-22

**Authors:** Yan Liang, Ni Wang, Qing Li, Huiyu Jiang

**Affiliations:** 1 Hubei Key Laboratory of Biomass Fibers and Eco-Dyeing & Finishing, School of Textile Science and Engineering, Wuhan Textile University, Wuhan 430200, China; 2215383012@mail.wtu.edu.cn; 2 School of Fashion, Wuhan Textile University, Wuhan 430200, China; wangni@wtu.edu.cn; 3 China National Textile and Apparel Council Key Laboratory of Natural Dyes, Soochow University, Suzhou 215123, China

**Keywords:** ink-jet printing, silk, gardenia blue, eco-friendly materials, hydrophobization

## Abstract

Ink-jet-printed silk, a premium textile material, was achieved by utilizing a bio-based gardenia blue dye. However, the sharpness of the printing pattern is difficult to control due to the limited water-retention capacity of silk. To address this issue, three polysaccharide derivatives, namely, sodium alginate (SA), low-viscosity hydroxypropyl methyl cellulose (HPMC-I), and high-viscosity hydroxypropyl methyl cellulose (HPMC-II), were employed as thickeners to modify the silk by the dipping–padding method. Firstly, the preparation of the gardenia blue ink and the rheology assessment of the thickener solution were conducted. Furthermore, the impacts of different thickeners on the micro-morphology, element composition, and hydrophilicity of the silk, along with the wetting behavior of the ink on the silk, were analyzed comparatively in order to identify an appropriate thickener for preserving pattern outlines. Lastly, the color features, color fastness, and wearing characteristics of the printed silk were discussed to evaluate the overall printing quality. Research results showed that the optimized ink formulation, comprising 12% gardenia blue, 21% alcohols, and 5.5% surfactant, met the requirements for ink-jet printing (with a viscosity of 4.48 mPa·s, a surface tension of 34.12 mN/m, and a particle size of 153 nm). The HPMC-II solution exhibited prominent shear-thinning behavior, high elasticity, and thixotropy, facilitating the achievement of an even modification effect. The treatment of the silk with HPMC-II resulted in the most notable decrease in hydrophilicity. This can be attributed to the presence of filled gaps and a dense film on the fibers’ surface after the HPMC-II treatment, as observed by scanning electron microscopy. Additionally, X-ray photoelectron spectroscopy analysis confirmed that the HPMC-II treatment introduced the highest content of hydrophobic groups on the fiber surface. The reduced hydrophilicity inhibited the excessive diffusion and penetration of gardenia blue ink, contributing to a distinct printing image and enhanced apparent color depth. Moreover, the printed silk demonstrated qualified color fastness to rubbing and soaping (exceeding grade four), a soft handle feeling, an ignorable strength loss (below 5%), and a favorable air/moisture penetrability. In general, the surface modification with the HPMC-II treatment has been proven as an effective strategy for upgrading the image quality of bio-based dye-printed silk.

## 1. Introduction

Silk, celebrated for its luxurious sheen and exceptional comfort on the skin, has long been revered as the “queen of fabrics” and has been extensively utilized in the textile industry for thousands of years [1,2]. According to data from 2019, silk production in China accounts for 63% of the world’s production of this fiber [3].

The most commonly used dyes for silk are reactive and acid dyes. Reactive dyes have a full color spectrum but a low fixation rate and a high effluent volume [4]. Acid dyes have a high fixation rate, but their wet rubbing fastness is poor [5]. Moreover, both dyes are synthetic dyes, which present a potential hazard in terms of safety and environmental protection. In particular, those dyes with benzidine structures represent a carcinogenic risk.

Plant dyes are derived from various parts of plants, such as the roots, leaves, and fruits, and are known for their non-toxic nature and eco-friendly properties [6]. In order to facilitate the sustainable development of the dyeing and printing of silk, using plant dyes instead of synthetic dyes has been widely considered. Curcumin was utilized in the production of printing ink, which had been instrumental in achieving an enhanced color intensity on silk, nylon, and wool fabrics [7]. Some researchers have studied the persistence and stability of natural indigo dye ink. The satisfactory printing performance of natural indigo ink was demonstrated by testing the color fastness of the printed textile [8]. A meticulous study has been conducted on the application of gardenia yellow in ink-jet printing on wool fabrics. The research indicated that wool fabric treated with tannins exhibited enhanced color fastness and antibacterial properties when printed with gardenia yellow [9].

**Figure 1:**
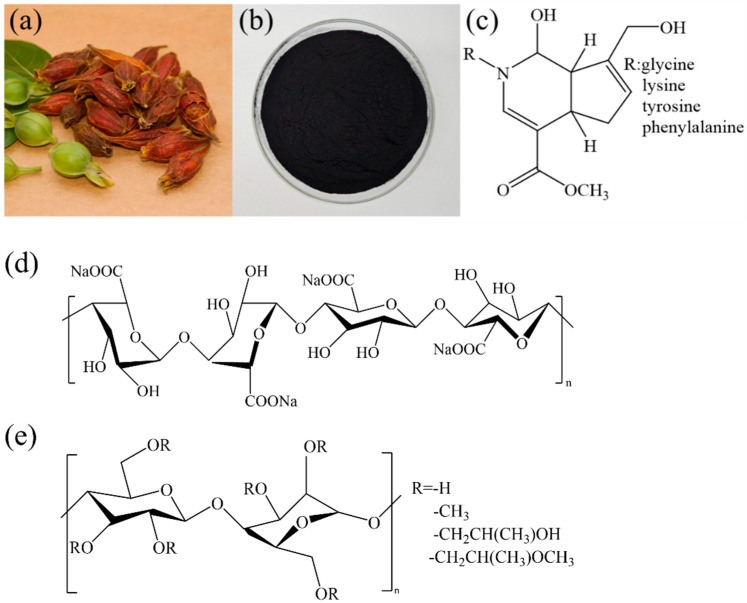
(**a**) Natural *Gardenia* plant, (**b**) gardenia blue dyestuff, (**c**) molecular formula of the dyestuff, (**d**) polymer formula of sodium alginate, and (**e**) hydroxypropyl methyl cellulose.

It is well known that obtaining blue dyes is the most challenging due to the scarcity of natural sources [10]. As shown in Figure 1a,b, a natural dark-blue dyestuff known as gardenia blue has been discovered to possess an equivalent coloring capacity to that of synthetic dyes, which can be obtained from a member of the cedar family—*Gardenia jasminoides* [11,12]. Compared to other natural dyes, it possesses significantly greater stability [11]. According to the chemical formula of gardenia blue (Figure 1c), the R group, i.e., glycine, lysine, tyrosine, or phenylalanine, possesses acidity because it contains a carboxyl group (-COOH) which can release protons (H^+^) [13]. As a result, the ionized gardenia blue exhibits electronegativity, thereby having a natural affinity toward the protonated terminal amino groups (-NH^3+^) in silk fibers via the ionic bonds under acidic conditions [14]. Therefore, this study proposes the use of gardenia blue as dyestuff to tackle the problems of unsafe and environmental hazards stemming from synthetic dyes for silk printing.

Printing is considered as the partial dyeing of a fabric. Digital ink-jet printing has surpassed traditional techniques such as roller and screen printing due to its superior pattern precision, flexible processing mode, and quick response to commercial demands [15]. Modern printed silk products possess intricate patterns and a wide range of colors. It has been difficult for traditionally printed silk to meet the personalized demands of consumers, leading to a shift towards digitally printed silk products. The combination of digital ink-jet printing with silk fabric can enhance the value of silk products significantly. For example, high-end fashion brands are increasingly incorporating unique designs into silk garments through ink-jet printing [16].

In comparison to natural fabrics such as cotton and woolen fabrics, silk fabrics are typically characterized by a high degree of thinness and lightness, and a distinctive porous structure [17,18]. Such structural features greatly limit the capacity of ink absorption, leading to weak ink-retention capabilities and the problem of light printing colors [19]. Additionally, it is easy for pronounced ink permeation to occur owing to the inadequate cohesion of ink droplets on the silk fabric’s surface [20]. To solve the above issues, it is essential to carry out the surface modification of silk fabrics using pastes, including thickeners, hygroscopic agents, dyeing accelerants, etc. [15]. Usually, this process is named sizing pretreatment, which creates a thin layer on the fabric’s surface. The thin paste layer functions as the carrier absorbing the ink to improve the printing color depth. In addition, the paste layer enhances the precision and outline of patterns by preventing the capillary action between the yarns, the diffusion of ink along the fibers, and the migration of dye during the drying process [21].

There are various thickeners used in the sizing pretreatment. Among them, cellulose derivatives are recognized for their widespread application as thickeners in ink-jet printing due to their weak affinity towards dyes, excellent rheological properties, and the advantage of being easily washed off [22]. For instance, wool fabrics treated with carboxymethyl hydroxypropyl cellulose (CMHPC) exhibit a significant enhancement in their ability to prevent the uncontrolled permeation of ink droplets, because CMHPC produces a continuous and thin film on the fabric [23]. The application of hydroxyethyl cellulose significantly enhances the color retention, depth, and brightness of flax fabric after ink-jet printing [24].

Hydroxypropyl methylcellulose (HPMC, Figure 1d) consists methyl and hydroxypropyl groups, which replace the hydroxyl group of cellulose in a certain proportion [25]. HPMC can be applied in the pretreatment of cotton and linen fabrics before ink-jet printing with reactive dyes. Research results demonstrated that the printing image quality was obviously upgraded because the HPMC film reduced the permeation of the ink towards to the fabric’s reverse side [25,26]. However, relevant reports focusing on the utilization of HPMC as the pretreatment thickener for the printing of silk fabrics are limited. Therefore, to solve the issues of light colors and unclear outlines in silk printing, two kinds of HPMCs with different structural viscosities were selected to pretreat silk fabrics through their surface modification. Moreover, sodium alginate (Figure 1d), which stands out as a prevalent printing thickener that is suitable for a variety of fabrics, was used as the comparative sample in this study.

## 2. Materials and Methods

### 2.1. Materials

Silk crepe (16 mm, 70 g/m^2^) was procured from Tallinn High-end Garment Fabrics Co., Ltd. (Haining, China). The gardenia blue used in this study was supplied by Hubei Kangleyuan Biotechnology Co., Ltd. (Qianjiang, China). Hydroxypropyl methyl cellulose (HPMC-I), characterized by a viscosity of 50 mPa·s, and sodium alginate (SA), with a viscosity of 200 mPa·s, were both procured from Shanghai Aladdin Biochemical Technology Co., Ltd. (Shanghai, China). Hydroxypropyl methyl cellulose (HPMC-II), characterized by a viscosity of 400 mPa·s, was sourced from Shanghai Macklin Biochemical Co., Ltd. (Shanghai, China). Urea, diethylene glycol, glycerol, ethanol, ethylene glycol, triethanolamine, acetic acid, and diiodomethane were of analytical reagent grade and supplied by Sinopharm Chemical Reagent Co., Ltd. (Shanghai, China). Triton X-100 and sodium dodecyl sulfate were chemically pure reagents and purchased from Sinopharm Chemical Reagent Co., Ltd. (Shanghai, China). The antifoam agent T303 was provided by Guangdong Demei Fine Chemical Co., Ltd. (Foshan, China). Deionized water was used exclusively in all of the experiments. The 0.45-micrometer filtration membrane was provided by Nanjing Good Quality Full Experimental Equipment Co., Ltd. (Nanjing, China).

### 2.2. The Preparation of Gardenia Blue Ink

**Figure 2:**
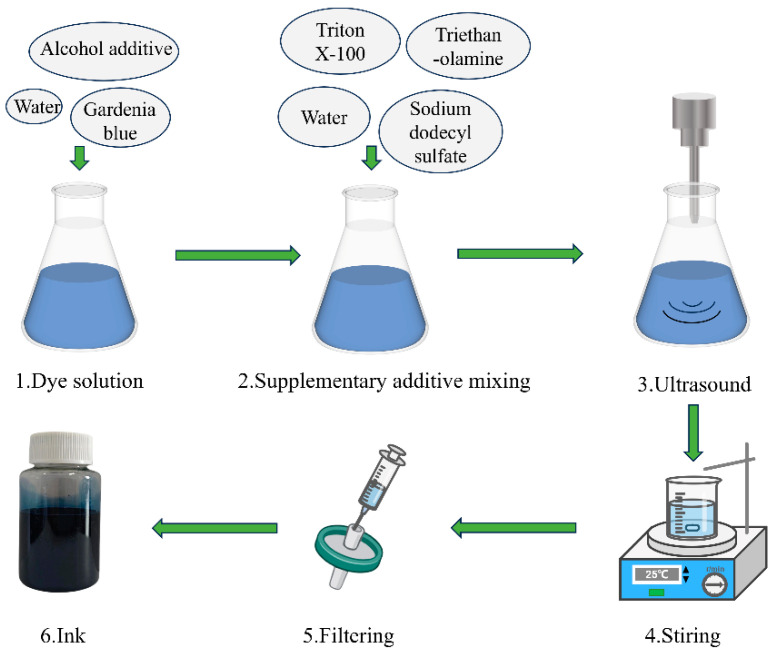
Preparation processes of gardenia blue ink.

As shown in Figure 2, firstly, different dosages of the gardenia blue dye (4–16 g) were dissolved in an aqueous solution (100 g) containing ethanol (10 g), ethylene glycol (10 g), and glycerol (1 g) with the aid of a YM-1000CT ultrasonicator (Shanghai Yu Ming Instrument Co., Ltd., Shanghai, China). The total power of the ultrasonic wave was 1000 W, and 70% of the power was applied. Afterwards, Triton X-100 (5 g), sodium dodecyl sulfate (0.5 g), and triethanolamine (0.25 g) were separately added into the prepared gardenia blue solution, which then underwent dispersal treatment by ultrasonication for 25 min. Finally, above-prepared solution was stirred by a magnetic stirrer (HJ-6A, Changzhou Chemical Energy Instrument Co., Ltd., Changzhou, China) for four hours and immediately filtered through a 0.45 μm filtration membrane.

### 2.3. The Preparation of Treatment Solution

The treatment solution mainly consisted of diethylene glycol, urea, thickener, and deionized water. In particular, urea (6 g) and diethylene glycol (4 g) were mixed with 88 g of deionized water to obtain a uniform solution. Subsequently, one kind of thickener (2 g), i.e., SA, HPMC-I, or HPMC-II, was gradually added to the above solution and then thoroughly stirred using the LC-OES-100SH mechanical stirrer (Shanghai Li-Chen Bang Xi Instrument Technology Co., Ltd., Shanghai, China) at 450 revolutions per minute for 30 min. Lastly, the pH value of the treatment solution was adjusted to 4 by acetic acid.

### 2.4. The Modification of Silk Fabric

The silk fabric was modified by the method of dipping–padding. Concretely speaking, the fabric was totally immersed in the treatment solution described in Section 2.3 and then padded using a rolling mill machine (YMPO-02S-630, Laizhou Yuanmao Instrument Co., Ltd., Laizhou, China). The dipping and padding operation was carried out in parallel twice. All of the modified silk fabrics were dried in a dryer at 65 °C for 3 min. The mass of the modified fabric was twice that of the original fabric. Untreated fabric, modified fabric using SA, modified fabric using HPMC-I, and modified fabric using HPMC-II were, respectively, labeled as Fabr.I, Fabr.II, Fabr.III, and Fabr.IV in this study.

### 2.5. Ink-Jet Printing Process

**Figure 3:**
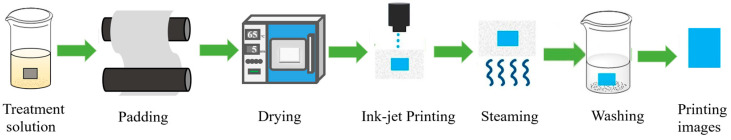
Schematic diagram of the ink-jet printing process.

As shown in Figure 3, an ink-jet printer (R330, Epson (China) Co., Ltd., Beijing, China) was used in this study. The gardenia blue ink was injected into the printer cartridge by a syringe. The modified silk fabric was printed by adopting the mode of “Quality Picture”. Afterwards, the printed silk was steamed in a steamer (RC-CSD2540, Shanghai Luozhong Technology Development Co., Ltd., Shanghai, China) at 102 °C for 20 min under saturated vapor conditions. When the steaming was finished, the printed samples were washed with cold water (20 °C), hot water (60 °C), and cold water (20 °C), in sequence. Finally, the printed samples were dried in the open air for the following tests.

### 2.6. Analytical Methods

#### 2.6.1. Physical and Chemical Properties of the Ink

The particle size and zeta potential of the ink were evaluated using a potential and size analyzer (Nano-ZS90, Malvern, UK). The apparent viscosity of the ink was tested by a rotational viscometer (NDJ-5S, Shanghai Li-ChenInstrument Technology Co., Ltd., Shanghai, China) at 25 °C using a No. 0 rotor with 60 revolutions per minute. The surface tension of the ink was measured by the platinum plate method using the fully automatic surface tension meter (SFZL-1, Shanghai Yingnuo Precision Instrument Co., Ltd., Shanghai, China). The conductivity of the ink was tested using a conductivity meter (DDS-30T, Shanghai Jinsi Magnetic Instrument Co., Ltd., Shanghai, China). Different concentrations of gardenia blue ink were diluted to obtain their spectra in the range of 400–700 nm using a UV–Vis spectrophotometer (TU-1950, Beijing Persee General Instrument Co., Ltd., Beijing, China).

#### 2.6.2. Rheological Properties of the Thickener Solution

A rheometer (AR2000, TA Company, New Castle, DE, USA) was used for the measurement of the rheological curves, viscoelastic properties, and thixotropies of the treatment solutions at 25 °C. The rheological curves were obtained by the method of steady-shear. The shear rate varied between 0.01 and 1000 s^−1^, and each point was scanned for 5 s. The equilibrium time was 30 s. The viscoelastic properties were measured by dynamic frequency scanning. The frequency range was 0.01–100 Hz with a fixed strain of 0.1%. The curves of the energy storage modulus (*G*′) and energy dissipation modulus (*G*″) of the treatment fluid with respect to the frequency were obtained. The loss angle (*δ*) was calculated by Equation (1). A thixotropy test was performed using a distributed shear method. The steady-shear was successively carried out at a fixed shear rate, i.e., 1 s^−1^, 400 s^−1^, and 1 s^−1^, for 120 s. All of the test samples contained only thickeners and water.

(1) $$\delta =DEGREES\left(ATAN\left(\frac{G^{\prime \prime }}{G^{\prime }}\right)\right)$$

#### 2.6.3. Hydrophilicity of Silk Fabrics

The dynamic contact angle of 3 μL of water droplets on the silk fabric was measured using a contact angle goniometer (OCA15EC, Dataphysics, Filderstadt, Germany). In accordance with AATCC 79-2010, the spread time of a water droplet was measured when the water droplet fell on the fabric surface until the reflective surface disappeared completely. All of the values were the averages of five measurements. The Washburn Function (see Equation (2)) was used to analyze the transverse capillary of the fluid inside the silk fiber, thereby further explaining the difference in the hydrophilicity.

(2) $$h^{2}=\frac{\gamma \cdot \sigma \cdot \cos\theta \cdot t}{2\beta }$$

where *h*, *r*, *σ*, *θ*, *t*, and *β* represent the diffusion distance, radius of the capillary when oriented horizontally, surface tension, contact angle, diffusion time, and viscosity of the fluid, respectively, during the process of fluid diffusion.

#### 2.6.4. SEM and XPS

A scanning electron microscope (Phenom, Eindhoven, The Netherlands) was used to observe the surface morphologies of silk fabrics before and after the modification. All of the samples underwent gold-spraying treatment before the test. The accelerating electronic voltage was 5 kv. The surface elemental alterations of the silk fibers were examined using an X-ray photoelectron spectroscopy apparatus (AXIS Ultra DLD X, Shimadzu, Kyoto, Japan). The wide range spectra from 0 to 1200 electron volts (eV) and the high-resolution narrow spectra for carbon elements were documented. An XPS instrument was calibrated as follows: the gold and copper standard samples were fed into the instrument after removing their oxidation layer to directly test Au 4f and Cu 2p. If the position and shape of their peaks were right, the XPS test of the silk samples were further carried out.

#### 2.6.5. The Infiltration Behavior of Ink Droplets

The diffusion of ink droplets on the surface of the fabric was tested according to the standard JIS L 1907-2010, Water Absorption Test Method for Textiles. The customary procedure was to determine the surface area produced by a droplet of 3 μL of ink released from a fixed distance once entirely diffused across the fabric.

Line widths of 0.35 mm were created by utilizing Photoshop software (Version 20.0.4). The printed fabrics were examined under a digital microscope (RX-100, HIROX, Tokyo, Japan). The line widths were measured randomly at three positions and then the average value of three widths was recorded.

#### 2.6.6. Color Features and Color Fastness

The color features of the fabric, i.e., the CIE a*, CIE b*, reflectance (*R*%), and color depth (*K*/*S*), were tested using a reflectance spectrophotometer (Datacolor, Lawrenceville, NJ, USA). The penetration ratio of the dyes (*P*) was calculated according to Equation (3). The soaping fastness of the printed silk fabrics were tested according to GB/T 3921-2008 which is equivalent to ISO 105-C10:2006,MOD. The rubbing fastness of the printed silk fabrics were tested according to GB/T 3920-2008 which is equivalent to ISO 105-X12:2001,MOD.The assessment of the soaping fastness and rubbing fastness was rated on five-grade scale, with grade 5 indicating superior performance and grade 1 representing poor performance.

(3) $$P=\frac{{\left(K/S\right)}_{b}}{0.5\times \left[{\left(K/S\right)}_{f}+{\left(K/S\right)}_{b}\right]}\times 100\%$$

where (*K*/*S*)*_f_* and (*K*/*S*)*_b_* indicate the color depth on the front side and back side of the printed fabric, respectively.

#### 2.6.7. Wearing Characteristics

The hand feeling of the silk fabric was tested by using a fabric touch tester (RF4008FTT, Shenzhen Ruifeng Instrument Co., Ltd., Shenzhen, China). The fabric was cut into an L-shape with a size of 310 mm × 310 mm.

The breaking strength of the silk fabric was tested using an electronic strength machine (YG065H, Laizhou Electronic Instrument Co., Ltd., Laizhou, China) according to GB/T 3916-2013 which is equivalent to ISO 2062:2009,MOD. The sample size was 50 mm × 300 mm.

The air permeability was tested by a fully automatic air permeability meter (YG461E-III, Wenzhou Fangyuan Instrument Co., Ltd., Wenzhou, China) according to GB/T 5453-1997 which is equivalent to ISO 9237:1995. The test pressure was 100 Pa and the test area was 20 cm^2^.

The moisture permeability was measured by a fully automatic moisture permeability tester (YG601H, Ningbo Textile Instrument Factory, Ningbo, China) according to GB/T 12704.2-2009 which is equivalent to ASTM E96/E96M-16.

## 3. Results

### 3.1. Physicochemical Properties of Gardenia Blue Ink

Ink-jet printers for textiles require an ink that adheres to a range of physical and chemical criteria. The apparent viscosity, surface tension, particle size, electrical conductivity, and pH value are the key evaluation indicators for a printing ink. Generally, the suitable viscosity ranges from 2 to 5 mPa·s [27]. The formation of an ink droplet is significantly influenced by the surface tension of the ink, which ranges from 21 to 48 mN/m [28]. In order to prevent the printer nozzle from becoming clogged, the ink’s particle size cannot exceed 200 nm [29]. The electrical conductivity, a property of the ink’s salt content, is another factor that leads to nozzle clogging. Thus, the conductivity is required to be below 10,000 µS/cm [30]. The pH value of the ink is usually in the neutral range so to avoid the corrosion of the ink-delivery channels [31].

A series of inks with different concentrations of gardenia blue were prepared to select the deepest and most printable ink. The physicochemical properties of the inks are displayed in Table 1. As seen from Table 1, the apparent viscosity and particle size of the ink increased with the increase in the concentration of gardenia blue. As the concentration increased, the distance between the dye molecules shortened and the internal friction between the molecules was improved, making the ink more viscous. The aggregation of dye molecules in the ink gradually enhanced as the concentration of the dye increased, resulting in the increase in the particle size. The decrease in the difference in the zeta potential also indicated the increased probability of the aggregation between the dyes at high concentrations [32].

In all, when the concentration varied in the range of 4–12%, the apparent viscosity and particle size met the demand of ink-jet printing. Additionally, all of the ink samples exhibited appropriate surface tension (32–36 mN/m), pH values (6.38–7.75), and electrical conductivity (<1322 µS/cm). Consequently, the employment of the ink containing 12% gardenia blue in the following experiment can be advantageous for a fluent process of ink-jet printing. Moreover, it is conducive to the prevention of nozzle clogging and the enhancement in the color depth of the printed fabric.

**Figure 4:**
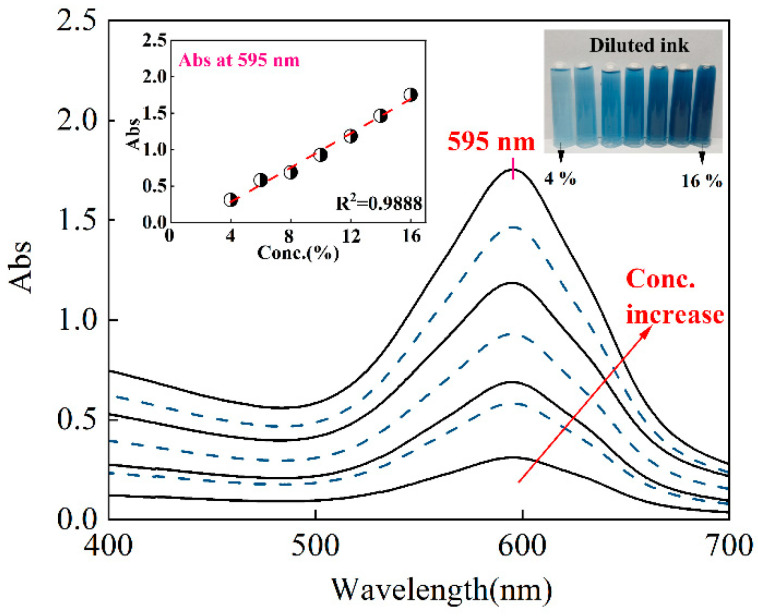
Absorption spectra curve and calibration curve of gardenia blue ink with various concentrations.

Figure 4 illustrates the absorption spectra of the gardenia blue ink across a range of concentrations. A distinct absorption peak was exhibited at the wavelength of 595 nm owing to the presence of a conjugated system consisting of several conjugated double bonds (Figure 1c). As the concentration of ink escalated, the absorption peak’s intensity correspondingly amplified, signifying a deepening of the ink’s color intensity. The absorption peak’s position remained relatively constant as the concentration of the dye increased, which supports the satisfactory solubility of the gardenia blue dye [9]. As seen in the inserted picture in Figure 4, the absorbance at 595 nm was linearly proportional to the dye’s concentration, with a correlation coefficient up to 0.9888, indicating the high purity of the used gardenia blue ink. In short, the result of the absorption spectra analysis proves the feasibility of adopting gardenia blue for the preparation of bio-based printing ink.

### 3.2. Rheological Properties of the Thickener Solution

**Figure 5:**
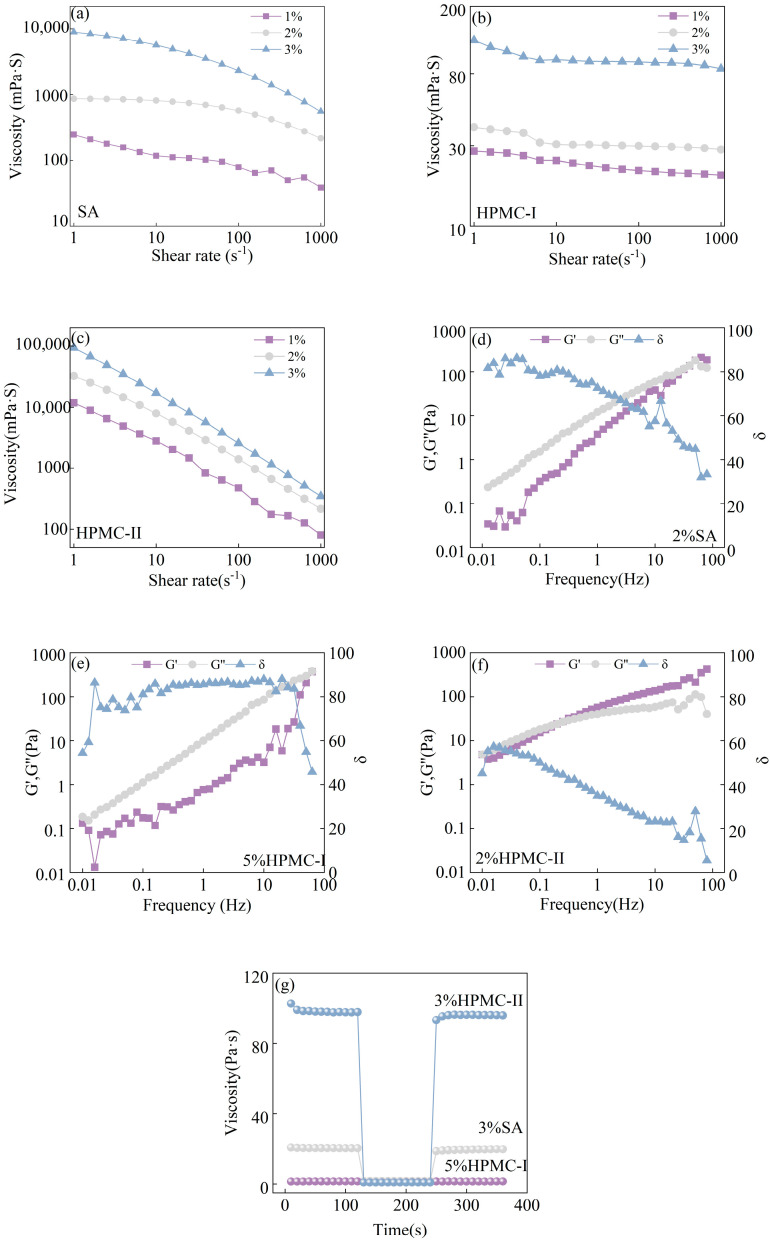
Steady-shear properties of the thickener aqueous solutions: (**a**) SA, (**b**) HPMC-I, and (**c**) HPMC-II; viscoelasticity of the thickener aqueous solutions: (**d**) SA, (**e**) HPMC-I, and (**f**) HPMC-II; (**g**) thixotropic properties of the SA, HPMC-I, and HPMC-II aqueous solutions.

Steady-state shear tests were carried out for the SA, HPMC-I, and HPMC-II aqueous solutions, and the results are shown in Figure 5a–c. With the augmentation of the shear rate, all of the thickener solutions exhibited shear-thinning behavior regardless of the thickener’s concentration. Among the three thickeners, the viscosity of the HPMC-II solution declined the most dramatically, while the HPMC-I and SA solutions demonstrated relatively inconspicuous reductions in viscosity. These phenomena proved that the HPMC-II solution exhibited the most obvious pseudoplastic fluid behavior. This is because HPMC-II possesses the highest structural viscosity compared to SA and HPMC-I, contributing to the greatest zero-shear viscosity. When the thickener solution is subjected to shear, the entanglements between the macromolecule chains are broken and they are orientated in the direction of the flow, leading to a reduction in the resistance to the flow, which is manifested as a decrease in the viscosity. The decline in viscosity during this process is more pronounced with an increasing shear rate [33].

Further, the viscoelasticity of above three solutions was measured and the results are illustrated in Figure 5d–f. For the HPMC-II solution, its energy storage modulus (*G*′) and loss modulus (*G*″) were almost equal when the frequency was less than 1 Hz. As the frequency continued to increase from 1 Hz to 100 Hz, the *G*′ became larger than *G*″. The tangent of the loss angle (*δ*) is the ratio of *G*″ to *G*′. It can be found that the *δ* of the HPMC-II solution was smaller than that of the SA and HPMC-I solutions, especially when the test frequency exceeded 1 Hz. These facts implied that the elastic behavior of the HPMC-II solution was more obvious than the viscous behavior. By contrast, both the SA and HPMC-I fluids mainly exhibited evident viscous behaviors, as demonstrated in Figure 5e,f.

Finally, the thixotropies of the SA, HPMC-I, and HPMC-II solutions were compared by the distributed shear method, and the results are shown in Figure 5g. The viscosity of the HPMC-II solution displayed a slightly decreasing trend when the shear rate was below 1 s^−1^. When the shear rate was suddenly increased up to 400 s^−1^, the viscosity of the HPMC-II solution decreased rapidly. When the shear rate was set to 1 s^−1^ again, its viscosity restored to the original level. Moreover, the HPMC-II solution showed the largest change margin in viscosity when compared to the SA and HPMC-I solutions, demonstrating the most significant thixotropy for the HPMC-II solution. This obvious thixotropy indicates that the polymer chains are capable of restoring their original entanglement structures rapidly when the external forces disappear [23]. Therefore, it was easy for the HPMC-II solution to recover its original physical state due to the hydrogen bonding and van der Waals forces existing between the molecular chains.

In summary, the HPMC-II solution had the most prominent characteristics of shear thinning, high elasticity, and thixotropy. The property of shear thinning made the penetration of the solution into the silk fabric more rapid during the dipping–padding process, as shown in Figure 3. The elastic behavior facilitated an easy separation of the excess treatment solution from the fabric, contributing to a uniform modification effect [23]. The high thixotropy meant that the treatment fluid transferred to the fabric quickly returned to its original viscosity after the padding process was completed, avoiding the excessive spread of the treatment fluid along a single yarn [23]. Therefore, it can be concluded that the HPMC-II solution is applicable for the modification treatment due to its more evident pseudoplasticity, elasticity, and thixotropy compared to those of the SA and HPMC-I solutions.

### 3.3. Surface Properties of the Modified Silk

#### 3.3.1. Micro-Morphology

**Figure 6:**
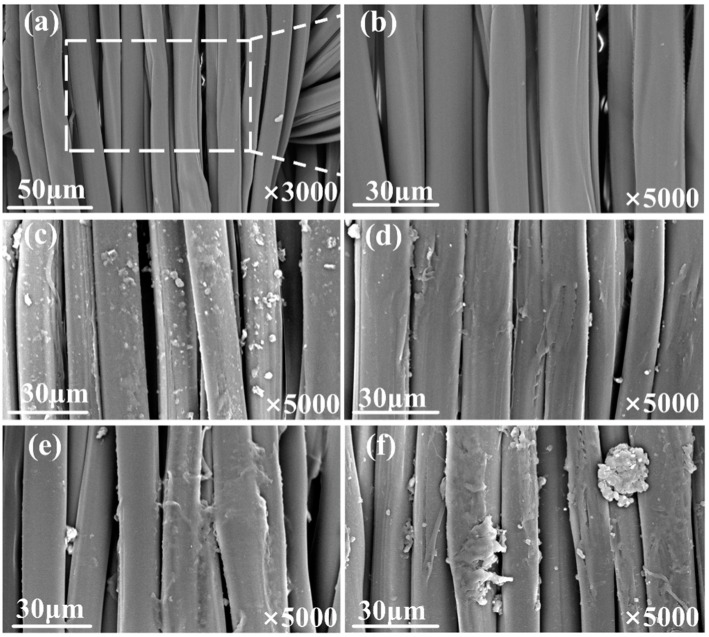
SEM images of differently treated silk fabrics: (**a**,**b**) Fabr.I; (**c**) thickener-free-treated fabric; (**d**) Fabr.II; (**e**) Fabr.III; (**f**) Fabr.IV.

The surface morphology of the silk fabric before and after the modification is shown in Figure 6. As seen in Figure 6a,b, some space existed at the intersection of the warp and weft yarns, and there were many gaps between the yarns in the same direction. Figure 6c displays the surface characteristic of the silk that underwent the treatment with the solution only containing urea, diethylene glycol, and water. Some white particles randomly distributed on the fiber’s surface are probably due to urea particles. Figure 6d–f depict the micro-morphology of the fabric treated using the SA, HPMC-I, and HPMC-II solutions, respectively. A partial blockage of the gaps between the yarns occurred on the above three samples, suggesting that the HPMC-II film formed in the yarn’s spaces. It is worth noting that the HPMC-II film exhibited a higher density when compared to the SA and HPMC-I films. This phenomenon is connected to the highest structural viscosity of HPMC-II observed in Figure 5c [20,34]. Therefore, the pretreatment obviously changed the morphology of the silk by filling the yarn gaps with thickener film, which further affected the diffusion and penetration of the ink in the subsequent printing process.

#### 3.3.2. Surface Element

**Figure 7:**
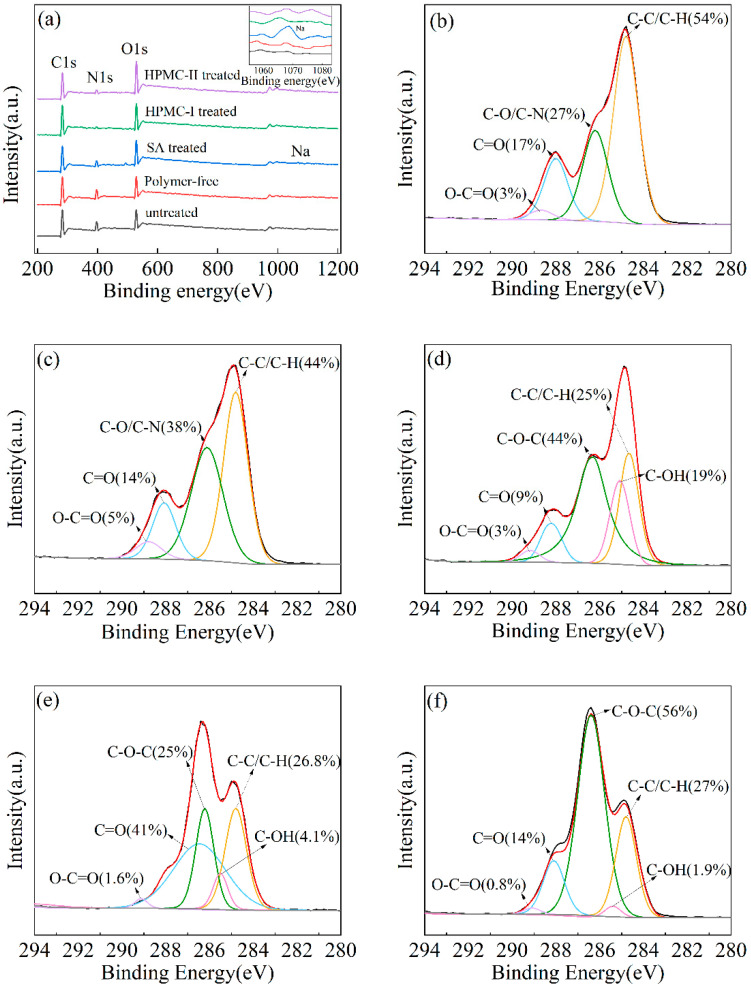
(**a**) XPS spectra of the differently treated fabrics; XPS spectra of the C1s of the differently treated fabrics: (**b**) Fabr.I; (**c**) polymer-free-treated fabric; (**d**) Fabr.II; (**e**) Fabr.III; (**f**) Fabr.IV.

The surface elemental compositions on the silk fabric, as determined through the XPS survey spectra, is illustrated in Figure 7. As shown in Figure 7a, the silk’s surface energy spectrum is primarily composed of elements such as carbon (C), oxygen (O), and nitrogen (N), which were the main constituents of the silk fiber itself. The surface energy spectrum of Fabr.II confirmed the presence of sodium (Na), which was provided by the SA. As exhibited in Figure 7b, in the high-resolution C1s spectra of the untreated Fabr.I, the following four primary types of carbon were identified: C-C/C-H, C-O/C-N, C=O, and O-C=O, with corresponding peak positions observed at 284.8 eV, 286.1 eV, 288.1 eV, and 289.1 eV, respectively. As shown in Figure 7c, the thickener-free fabric exhibited a slight increase in the content of C-O/C-N bonds, which may be attributed to the existence of urea crystals on its surface. As shown in Figure 7d–f, the content of the hydrophobic group C-C/C-H on Fabr.IV was the highest and surpassed that of Fabr.II and Fabr.III. Additionally, Fabr.IV possessed the lowest number of hydrophilic groups, including O-C=O and C-OH. Therefore, the silk achieved the most significant hydrophobic surface modification based on HPMC-II when compared to SA and HPMC-I, which can be attributed to the plentiful number of hydroxypropyl and methyl groups in HPMC-II.

### 3.4. Hydrophilicity of Silk Fabrics

**Figure 8:**
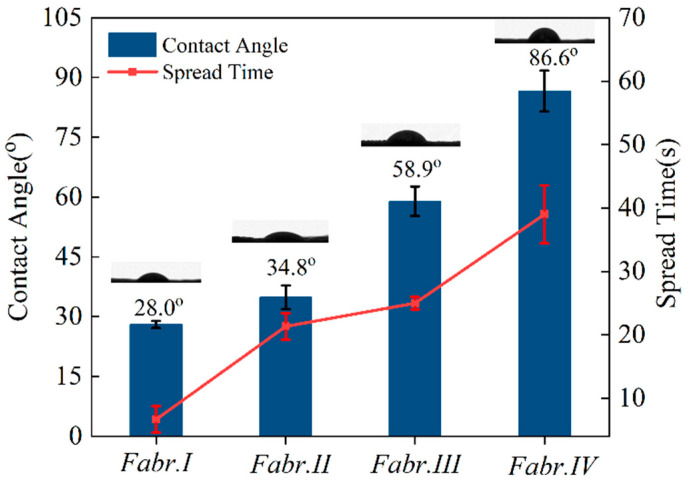
Hydrophilicity of differently treated silk fabrics (the error bar means the standard deviation).

Figure 8 displays the hydrophilicity of Fabr.I, Fabr.II, Fabr.III, and Fabr.IV. The droplet contact angle on the surface of Fabr.I was 28.0°. Meanwhile, the spread time of a water droplet on Fabr.I was 6 s, meaning that the untreated fabric had a hydrophilic surface which can be wetted immediately. In terms of the treated fabrics, their contact angles and spread times all increased. This is mainly because the yarn’s gaps in the treated fabric became smaller. Among the treated fabrics, Fabr.IV exhibited the maximum contact angle of 86.6° and the longest spread time of 39 s, indicating the most obvious hydrophobicity of the fabric treated by HPMC-II, followed by HPMC-I and SA, owing to the fact that the HPMC-II film was the densest, as observed in Figure 6f. Moreover, the higher structural viscosity of HPMC-II compared to HPMC-I indicates a higher degree of substitution of methyl and hydroxypropyl groups in HPMC-II. At the same time, SA lacks hydrophilic groups. Consequently, HPMC-II exhibited significant hydrophobicity when compared to the SA and HPMC-I, which is another explanation for the more obvious hydrophobicity of Fabr.IV. Additionally, the less significant standard deviation of the contact angle and spread time indicates that the hydrophilicity of the modified fabrics is relatively uniform.

As shown in Figure 8, Fabr.IV exhibits a contact angle of 86.6°. According to Equation (2), the spreading distance (*h*) of the water droplet on Fabr.IV was close to zero, indicating that the water droplet basically did not spread on Fabr.IV. Additionally, when compared with Fabr.I, Fabr.II, and Fabr.III, Fabr.IV possessed the lowest surface energy, as shown in Table S1, meaning that the membrane formed by HPMC-II on the silk fabric was the least hydrophilic [35]. Based on the analyses of the Washburn Function and the surface energy, it can be concluded that the spreading of water droplets along the capillary between the yarns was significantly inhibited, and the surface energy of the fabric decreased correspondingly, bringing about the most hydrophobicity of the treated fabric in HPMC-II.

### 3.5. The Infiltration Behavior of Ink Droplets

**Figure 9:**
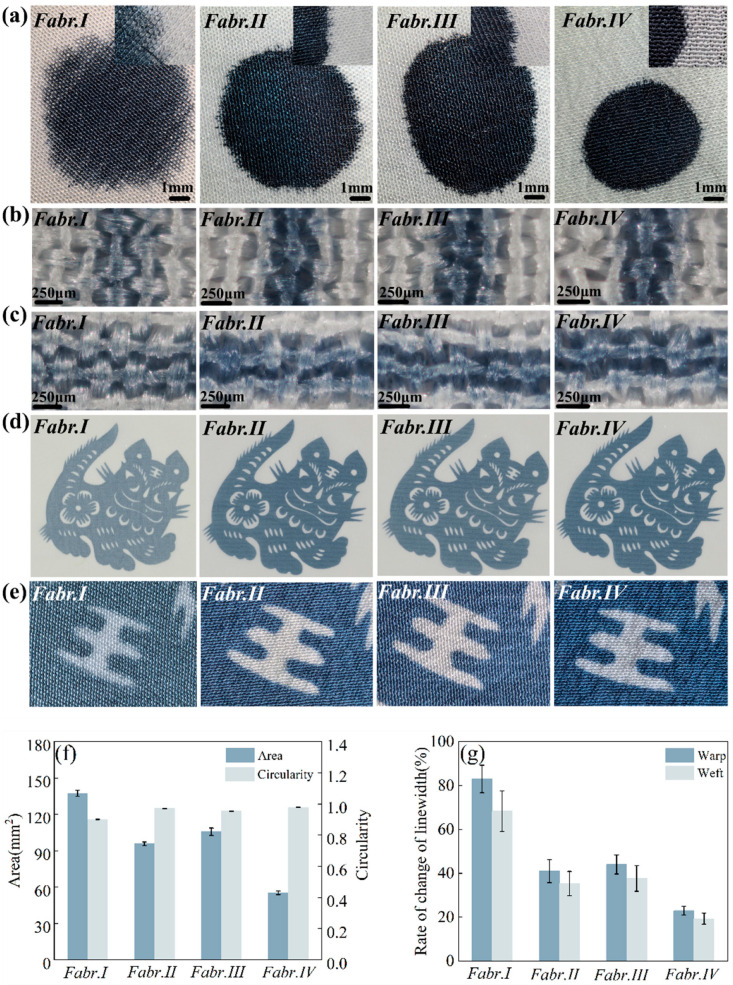
Shapes of ink droplet spread on differently treated fabrics (**a**); micrographs of the ink-jet-printed lines on differently treated fabrics in the warp (**b**) and weft (**c**) directions; ink-jet-printed patterns (**d**) and corresponding detailed pictures (**e**) on differently treated fabrics; the diffusion area and circularity of ink droplets (**f**); the change rate in the line width in the warp and weft directions (**g**) (the error bar means the standard deviation).

Figure 9a presents the shape of 6 μL ink droplets as they were placed on differently treated silk fabrics, and Figure 9f illustrates the spreading areas and the circularity of these ink droplets. The droplets on Fabr.I formed an approximate square and showed the largest spreading area, indicating that the ink simultaneously diffused. Thus, the fabric without treatment cannot inhibit the overspreading of ink droplets, leading to a poor definition. By contrast, the ink droplets on Fabr.II and Fabr.III displayed oval patterns, whereas a round-shaped pattern with the highest circularity of the ink droplets was eventually observed on Fabr.IV. The inset images in Figure 9a also show the obviously improved sharpness of the ink droplets when using HPMC-II. These facts prove that HPMC-II has been demonstrated to play a crucial role in enhancing the sharpness of ink droplets, with SA and HPMC-I following in significance.

Furthermore, the line image with the setting width of 400 µm was ink-jet printed on the fabric, and the printing results are shown in Figure 9b,c. The line was light in color, and the edge of the line was blurred, with severe bleeding on the untreated fabric in both the wrap and weft directions. An enhancement in the color depth can be observed on the treated fabrics. Moreover, the smallest change rate in the line width is shown on the HPMC-II-treated fabric in Figure 9g, demonstrating the best effect of HPMC-II in maintaining the pattern’s outline during the ink-jet printing. Additionally, the line width at different positions exhibited a less significant difference.

Moreover, a “tiger” image was printed on the different fabrics, and the printing results are shown in Figure 9d. It can be seen from Figure 9d that the printing pattern of Fabr.IV had the darkest coloration. Enlarged images of the tiger’s eyebrows in Figure 9e prove that the pattern outline of the printed Fabr.IV was the sharpest. According to the results above, it was found that HPMC-II demonstrated the most pronounced role in keeping the pattern’s sharpness and enhancing the color depth. This is mainly ascribed to the fact that HPMC-II formed a dense film on the fiber surface. Specifically, this film not only effectively reduced the spread of ink droplets along the wrap and weft fibers, but also inhibited the penetration of ink from the front side towards to the back side of the fabric.

### 3.6. Printing Performance and Mechanism

#### 3.6.1. Color Features and Color Fastness

**Figure 10:**
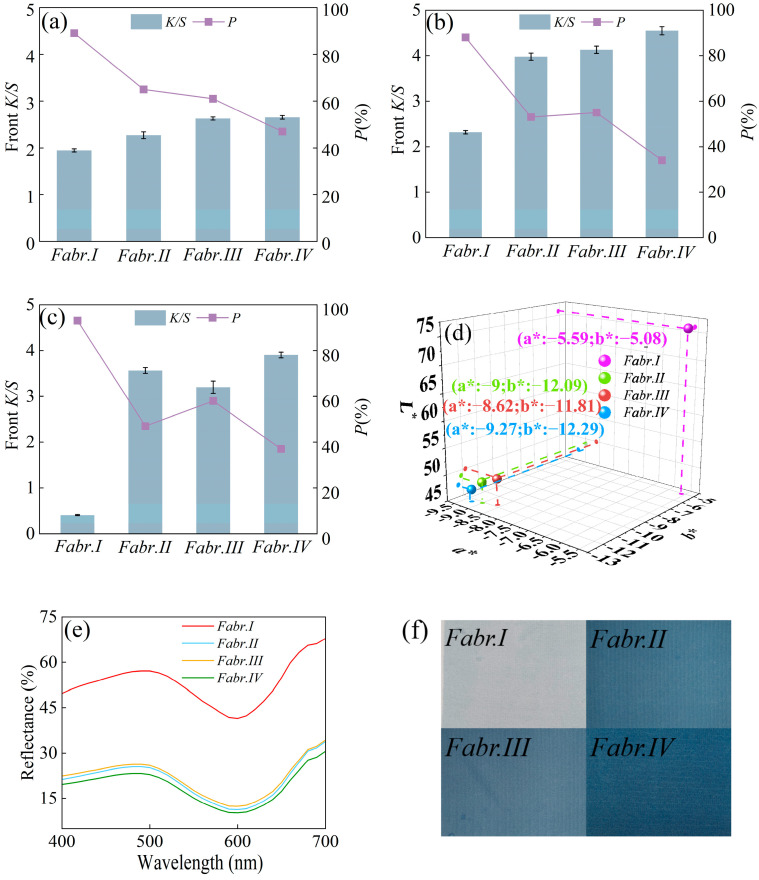
Front color depth and penetration ratio of gardenia blue for differently treated fabrics: (**a**) printed but not steamed fabrics; (**b**) steamed but not washed fabrics; (**c**) steamed and washed fabrics; CIE color coordinates (**d**); reflectance curves (**e**); photos of washed and printed Fabr.I, Fabr.II, Fabr.III, and Fabr.IV (**f**) (the error bars mean the standard deviation).

The apparent color depth (front *K*/*S*) and dye penetration ratio (*P*) of the printed fabrics were measured to assess the printing performance of differently treated silks. As shown in Figure 10a, the untreated silk had the highest penetration rate as compared to the other treated samples. Silk fabric, due to its high hydrophilicity, allows ink droplets to rapidly seep into the reverse side by capillary action. As a result, the untreated fabric exhibited the highest dye penetration ratio [15]. In comparison to Figure 10a, all of the treated fabrics shown in Figure 10b exhibited an obviously improved color depth after the steaming. This is because the steaming process (see Figure 3) can provide heating and moisture, which allows the gardenia blue molecules to dissolve and penetrate into the interior of the fibers, further forming physical and chemical bonds between the dye and silk substrate [36]. Additionally, the moisture absorbent, such as urea in the treatment solution, accelerated the dissolution of gardenia blue, and an acid medium (with a pH of 4) provided by acetic acid was conducive to the increased fixation ratio of the dyes [37].

As shown in Figure 10c, after the washing process, the lightest color for the printed Fabr.I indicates that the gardenia blue nearly cannot combined with the untreated silk, demonstrating that the printing depth can be enhanced effectively after the pretreatment. In particular, the printed Fabr.IV displayed the highest front *K/S* and lowest penetration ratio after steaming and washing. This is mainly due to the dense and hydrophobic HPMC-II film on the surface of the fiber, which is beneficial to the preservation of most of the ink at the front of the fabric.

As shown in Figure 10d, the smallest L* value for the printed Fabr.IV also proves that Fabr.IV can generate the deepest printing color. Both the absolute values of a* and b* of Fabr.IV reached the maximum, illustrating that the most vivid color and highest saturation had been obtained by Fabr.IV. Meanwhile, the lowest reflectance and deepest visible color of the printed Fabr.IV are displayed in Figure 10e and Figure 10f, respectively. These phenomena are consistent with the result shown Figure 10c. Additionally, the uniform color exhibited in Figure 10f is also consistent with the small standard deviation bars in Figure 10c.

Table 2 shows that all of the samples had satisfactory color fastness, including rubbing and washing, with the numerical levels exceeding three, meaning that the color fastness of the printed fabric was seldom influenced by the kind of thickener. Moreover, both the rubbing and washing fastness of the printed Fabr.IV reached up to a level of four or higher. Based on the results of the color feature and color fastness, it can be concluded that the deepest apparent color, lowest dye penetration, and qualified fastness can be attained for the silk through surface modification using HPMC-II. This is primarily attributed to the more obvious inhibition of ink diffusion when using HPMC-II compared to the other two thickeners.

#### 3.6.2. Mechanism of Printing

**Figure 11:**
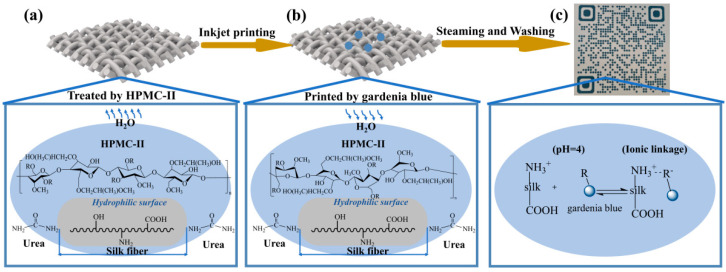
The function of HPMC-II to improve the performance of the ink-jet printing of silk using gardenia blue. (**a**) Drying process after HPMC-II treatment; (**b**) Steaming process after ink-jet printing; (**c**) Dye and fibre binding mechanism.

As shown in Figure 11a, the drying process of the modified fabric (see Figure 3) facilitated the movement of water from the fabric’s interior to its exterior, leading to a gradient in the moisture content for the HPMC-II film. The hydrophilic groups on the side chains of HPMC-II tended to face the outside of the HPMC-II film, and the moisture content on the surface of the HPMC-II paste was higher than that in the interior. The methyl and hydroxypropyl groups in HPMC-II covered the silk and made the surface of the silk hydrophobic. As shown in Figure 11b, during the process of steaming, water vapor came into contact with the urea and then entered the membrane. The water then moved from the outside to the inside, and the water content inside the paste was greater than that outside the surface, resulting in the distribution of the hydrophilic groups of HPMC-II towards the surface of the fiber. As shown in Figure 11c, the dye molecules approached the fibers with the help of water vapor and fixed onto the silk fibers mainly by electrostatic interactions [25], thereby finishing the whole printing process.

#### 3.6.3. Wearing Characteristics

Fabr.I was used as the control sample to determine the surface and bending properties to evaluate the effects of the modification and printing on the hand feeling of the fabric. BARa is the average stiffness of the flexural force in the warp direction and BARe is the average stiffness of the bending force in the weft direction. The lower value of BARa and BARe indicated the superior softness and elasticity of the fabric [38]. SFCa and SFCe are the surface friction coefficients in the warp and weft directions, respectively. SRWa and SRWe are the surface roughness wavelengths in the warp and weft directions, respectively. The smaller the above values, the smoother the feeling of the fabric [39].

**Figure 12:**
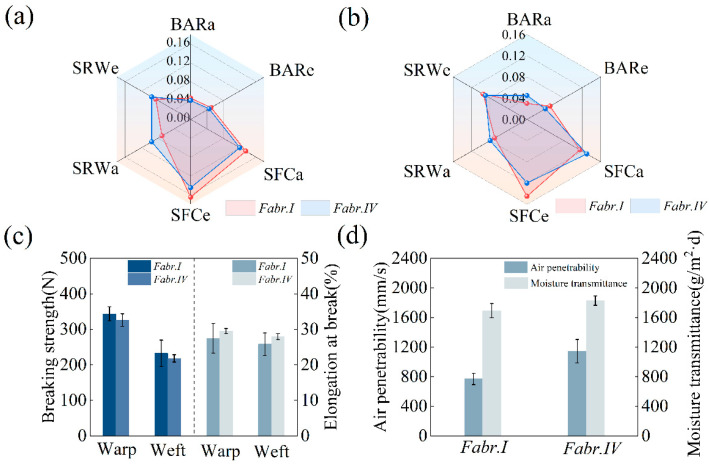
Surface and bending properties of Fabr.I and printed Fabr.IV in the warp (**a**) and weft (**b**) directions; mechanical properties (**c**), and air penetrability and moisture transmittance (**d**) of Fabr.I and printed Fabr.IV (the error bars mean the standard deviation).

As illustrated in Figure 12a, the printed Fabr.IV showed little difference in BARa, BARe, SFCa, SFCe, SRWa, and SRWe when compared with Fabr.I, demonstrating that there was no evident change in the softness, elasticity, and smoothness of the fabric. A similar phenomenon in the weft direction can be observed in Figure 12b. Therefore, the hand feeling of the silk did not decrease after the modification and printing.

The comparisons of the chemical properties, air permeability, and moisture transmittance of Fabr.I and the printed Fabr.IV are shown in Figure 12c,d. The breaking strength of the printed Fabr.IV decreased slightly in both the warp and weft directions, indicating that only slight damage occurred on the fabric (Figure 12c). The minor standard deviation bars mean that the difference in the breaking strength and the elongation in the three tests are less significant. The air permeability of the printed Fabr.IV was improved when compared to Fabr.I (Figure 12d). The breathability of a fabric depends mainly on the size and number of voids in the fabric, as well as on the structural parameters of the fabric (the thickness, fluffiness, etc.), which means that the whole printing process may have some influence on the fabric structure of silk. The moisture permeability of the printed Fabr.II did not change obviously compared to Fabr.I.

## 4. Conclusions

Silk fabric was treated with a modification solution containing urea, diethylene glycol, and one kind of polysaccharide derivative, i.e., SA, HPMC-I, or HPMC-II, resulting in the formation of a uniform and compact polymer film on the surface of the fibers. Gardenia blue as a bio-based dye was then applied to prepare an environmentally friendly printing ink. Subsequently, the modified fabric was subjected to ink-jet printing, steaming, and washing processes, successively. Ultimately, the silk fabric printed with gardenia blue exhibited the most vibrant apparent color and sharpest pattern outline after the modification with HPMC-II.

The research findings showed that the ink containing 12% gardenia blue met the requirements for ink-jet printing based on the examination of the viscosity, surface tension, and particle size of the ink. The HPMC-II solution demonstrated a uniform and superior modification effect due to its more evident pseudoplasticity, elasticity, and thixotropy compared to the SA and HPMC-I solutions. The most notable hydrophobicity was observed by the HPMC-II-modified silk because of the generation of a dense polymer film on the fiber surface, as revealed through SEM. Additionally, the increased content of the hydrophobic group C-C/C-H, as proven by the XPS analysis, was another reason. The application of HPMC-II effectively inhibited the spread of gardenia blue ink along the warp and weft fibers, as well as the penetration from the front to the back side of the fabric. When compared to the pretreatment using SA and HPMC-I solutions, the utilization of the HPMC-II solution achieved the deepest coloration, lowest dye penetration, and most satisfactory fastness (>four) for the printed silk. There were no significant alterations observed in the hand feeling, mechanical properties, air permeability, and moisture permeability for the printed fabric. In all, this research provided an effective and eco-friendly strategy to upgrade the image quality of ink-jet printing using bio-based dyes on silk fabric. In a future study, the combination of clean surface modification technology (e.g., plasma treatment) and sizing modification is planned for a further improved printing performance.

## Figures and Tables

**Table 1:** Physicochemical indices of the prepared gardenia blue ink.

Conc. of Gardenia Blue (%)	Apparent Viscosity (mPa·s)	Surface Tension (mN/m)	pH Value	Electrical Conductivity (μS/cm)	Partical Size (nm)	Zeta Potential (mV)
4	2.71	32.88	7.75	968	107.6	−33.3
6	3.12	33.02	7.35	1099	104.4	−19.7
8	3.16	33.04	6.92	1160	149.8	−23.7
10	3.97	33.97	6.78	1234	122.5	−22.1
12	4.48	34.12	6.54	1258	153.8	−20.7
14	5.39	35.63	6.38	1273	198.3	−16.1
16	6.26	36.26	6.67	1322	220.8	−14.4

**Table 2:** The washing fastness and rubbing fastness of the printed Fabr.II, Fabr.III, and Fabr.IV.

Samples	Rubbing Fastness		Washing Fastness		
			Color Staining		Color Change
	Dry	Wet	Silk	Cotton	
Fabr.II	5	4–5	5	5	4
Fabr.III	5	4–5	5	5	3
Fabr.IV	5	4–5	5	5	4

## Data Availability

The original contributions presented in the study are included in the article/Supplementary Material, further inquiries can be directed to the corresponding authors.

## Supplementary Materials

The following supporting information can be downloaded at: https://www.mdpi.com/article/10.3390/ma17143611/s1, Table S1: Contact angles and surface energy of silk fabrics.
