# Supplementary material for: Surface Modification of Silk Fabric by Polysaccharide Derivatives towards High-Quality Printing Performance Using Bio-Based Gardenia Blue Ink
Source: Materials (Basel). 2024 Jul 22;17(14):3611. doi: 10.3390/ma17143611 (PMC11278700; doi:10.3390/ma17143611)
Supplement: Supplementary file 1 [file materials-17-03611-s001.zip › materials-3091063-supplementary.pdf]

materials

MDPI

Supplementary Material for

Surface Modification of Silk Fabric by Polysaccharide Derivatives towards High-Quality Printing Performance Using Bio-Based Gardenia Blue Ink

Yan Liang [1], Ni Wang [2], Qing Li [1,3,*], Huiyu Jiang [1,*]

[1] Hubei Key Laboratory of Biomass Fibers and Eco-Dyeing & Finishing, School of Textile Science and Engineering, Wuhan Textile University, Wuhan 430200, China; 2215383012@mail.wtu.edu.cn

[2] School of Fashion, Wuhan Textile University, Wuhan 430200, China; wangni@wtu.edu.cn

[3] China National Textile and Apparel Council Key Laboratory of Natural Dyes, Soochow University, Suzhou 215123, China

* Corresponding: liqing@wtu.edu.cn (Q.L.); huiyujiang@wtu.edu.cn (H.J.)

Analysis of surface energy

Both the surface energy of the solid, represented by γ_S, and the surface tension of the liquid, denoted by γ_L, consist of two components: the dispersive components γ_S^d and γ_L^d, respectively, as well as the polar components γ_S^P and γ_L^P, respectively. θ_{water} and $\theta_{diiodomethane}$ present the contact angle of distilled water and diiodomethane on different silk fabrics, respectively. The data of contact angle were measured using a contact angle goniometer (OCA15EC, Dataphysics, Germany), as shown in Table S1. Subsequently, γ_s^d and γ_s^p were calculated according to equation (S1) and the results are also listed in Table S1. Lastly, the surface energy (γ_s) of differently treated silk fabrics were obtained according to equation (S2).

Table S1. Contact angles and surface energy of silk fabrics

Samples	Contact angles (°)		Surface energy (mJ/m^2)		
	θ_{water}	$\theta_{diiodomethane}$	γ_s^d	γ_s^p	γ_s
Fabr.I	28.0	43.8	17.30	47.10	64.40
Fabr.II	34.8	68.9	11.69	49.55	61.24

Fabr.III	58.9	74.5	3.44	30.30	33.74
Fabr.IV	86.6	113.0	1.47	21.22	22.69

$$\frac{\gamma_L(cos\theta + 1)}{2} = \sqrt{\gamma_S^d + \gamma_L^d} + \sqrt{\gamma_S^p + \gamma_L^p} \quad \text{(S1)}$$

$$\gamma_S = \gamma_S^d + \gamma_S^p \quad \text{(S2)}$$

In Table 2, *Fabr.II* exhibited a decrease in the dispersion component and an increase in the polar component when compared to *Fabr.I*. The formation of a smooth film on the fabric surface, coupled with the collective impact of hydrophilic groups such as -COONa and -OH, may account for the cause for this phenomenon [1]. Moreover, the contact angles of distilled water and diiodomethane on *Fabr.IV* were larger than those of *Fabr.I, Fabr.II, and Fabr.III,* and *Fabr.IV* displayed the lowest surface energy. Above results indicated that the hydrophilicity of the *Fabr.IV* was reduced markedly because of the presence of the hydrophobic chain provided by HPMC-II on the surface of *Fabr.IV*.

References

[1] Li M, Zhang L, An Y, et al. Relationship between silk fabric pretreatment, droplet spreading, and ink-jet printing accuracy of reactive dye inks. *J. Appl. Polym. Sci.* **2018**, *135*, 46703.
